# MiR‐483‐3p improves learning and memory abilities via XPO1 in Alzheimer's disease

**DOI:** 10.1002/brb3.2680

**Published:** 2022-07-14

**Authors:** Gang Luo, Xiaoyan Wang, Changya Liu

**Affiliations:** ^1^ Department of Rehabilitation Gezhouba Central Hospital of Sinopharm, The Third Clinical Medical College of China Three Gorges University Yichang Hubei China; ^2^ Department of Rehabilitation The Central Hospital of Wuhan, Tongji Medical College, Huazhong University of Science and Technology Wuhan Hubei China; ^3^ Department of Neurology Hubei Provincial Hospital of Traditional Chinese Medicine Wuhan Hubei China

**Keywords:** Alzheimer's disease, homocysteine, miR‐483‐3p, PC12 cells, XPO1

## Abstract

**Introduction:**

Alzheimer's disease (AD), a common form of dementia, has been reported to influence 27 million individuals globally. Several risk factors including oxidative stress, gut microbiota imbalance, and cognitive activity are reported to be closely associated with the initiation or progression of AD. Although miR‐483‐3p was identified to be downregulated in AD patient serum. However, the biological role and mechanism of miR‐483‐3p remained unknown in AD. Here, we explored the role of miR‐483‐3p in AD.

**Methods:**

Sprague–Dawley rats were injected with homocysteine (Hcy) to establish an AD animal model. The Morris water maze tests and contextual fear tests were conducted to assess the cognitive and memory abilities of rats. TUNEL staining was utilized to determine cell apoptosis. Luciferase reporter assay was used to evaluate the binding relation between miR‐483‐3p and exportin 1 (XPO1).

**Results:**

Homocysteine treatment (400 μg/kg) induced the learning, cognitive and memory defects of rats. miR‐483‐3p was downregulated in Hcy‐treated rat hippocampus. Functionally, miR‐483‐3p alleviated cell apoptosis and impairments of learning and memory abilities in Hcy‐treated rats. In addition, miR‐483‐3p inhibited cell apoptosis and protein level of AD‐associated factors (APP, BACE1, and Aβ1‐42) in PC12 cells. In mechanism, miR‐483‐3p was confirmed to target XPO1 in PC12 cells. XPO1 displayed high level in rat hippocampus and was negatively correlated with miR‐483‐3p levels. Finally, XPO1 overexpression rescued the suppressive effect of miR‐483‐3p on cell apoptosis and protein levels of AD‐associated factors.

**Conclusions:**

miR‐483‐3p alleviates neural cell apoptosis and impairments of learning and memory abilities by targeting XPO1 in AD.

## 1 INTRODUCTION

Alzheimer's disease (AD), a common form of dementia, is a leading cause of disability and poor health (morbidity) and characterized by a significant decline in cognitive and memory abilities (“2020 Alzheimer's disease facts & figures,” 2020). The population of Americans older than 64 with AD is projected to increase from 56 million in 2020 to 88 million by 2050 (“2020 Alzheimer's disease facts & figures,” 2020; “2021 Alzheimer's disease facts & figures,” 2021). Data from the World Health Organization showed that AD is about to affect 82 million patients in 2030 (“2020 Alzheimer's disease facts & figures,” 2020). Known risk factors including family history, aging, head injury, neuroinflammation, depression, oxidative stress, environmental metals exposure, gut microbiota imbalance, and cognitive activity are reported to be closely associated with the initiation or progression of AD (Briggs et al., 2016; Hodson, 2018; Lane et al., 2018; Mangialasche et al., 2010; Mantzavinos & Alexiou, 2017; Oboudiyat et al., 2013; Weller & Budson, 2018).

AD is accompanied with two pathologies: one is extracellular deposits of β‐amyloid (Aβ) plaques and another one is hyperphosphorylation of tau, a microtubule‐associated protein that leads to intracellular formation of neurofibrillary tangles in the brain (Wei et al., 2020b). Many biomarkers, such as amyloid precursor protein (APP), β‐site APP‐cleaving enzyme 1 (BACE1), Aβ peptide 1–42 (Aβ1‐42), and tau‐5, are associated with AD progression (Weng et al., 2020). Aβ protein is produced by protease cleavage of the type I transmembrane APP (Guo et al., 2020). It was proposed that the extracellular domain of APP causes cell‐to‐cell adhesion to facilitate synaptic connections (Ludewig & Korte, 2016). APP processing was dependent on three proteolytic secretase enzymes include α‐secretase, β‐secretase, and γ‐secretase. Specifically, α‐secretases refer to ADAM9, ADAM10, and ADAM17. BACE1 is the major β‐secretase in brain, and γ‐secretases include presenilins, niacstrin, PEN2, and APH1 (Guo et al., 2020; Zhang et al., 2011). APP cleavage generates amyloid peptides including Aβ37, 38, 39, 40, 42, and 43 (Guo et al., 2020). Aβ accumulates within neurons in both human AD cases and transgenic AD rodent models (Shie et al., 2003). Among these peptides, Aβ40 and Aβ42 are two major Aβ species in the brain, and soluble Aβ40 is more abundant than soluble Aβ42. Dysregulated APP processing can result in AD pathogenesis by increasing Aβ production (Guo et al., 2020). In this study, Aβ1‐40 was used to treat rat pheochromocytoma cells (PC12 cells). Aβ1‐40‐stimulated PC12 cells have been widely used for studies on AD‐associated neural injuries (Feng et al., 2013; Hu et al., 2021; Qian et al., 2015). According to other hypotheses, the pathogenesis of AD is also associated with oxidative stress, mitochondrial dysfunction, or neuroinflammation (Anand et al., 2014; Yang et al., 2020).

Homocysteine (Hcy) is a homolog of cysteine that is an intermediate form in the process of methionine metabolism. Based on previous experimental studies, Hcy induces molecular and cellular oxidative injury through reactive oxygen species (Kaplan et al., 2020). Moreover, Hcy toxicity is characterized by impairment of epigenetic control mechanisms of gene expression, such as DNA methylation, noncoding RNA, and histone modification (Perła‐Kaján & Jakubowski, 2019). Furthermore, Hcy can affect functions and structure of proteins by interacting with their cysteine or lysine residues (Kaplan et al., 2020). The lack of enzymes and vitamins implicated in Hcy metabolism and some pathological conditions result in increased blood plasma concentrations of Hcy, called hyperhomocysteinemia (HHcy) (Kaplan et al., 2020). HHcy has been reported in patients with AD, Parkinson's disease, heart failure, and cardiovascular disease (dos Santos et al., 2009; Nygård et al., 1995; Seshadri, 2006; Strauss et al., 2017).

Central nervous and cardiovascular system are closely interconnected. Specifically, cardiovascular systems can serve as a senor and effector for the central nervous system, in which the neurons strongly control cardiovascular functions (Critchley et al., 2018; Yang et al., 2020). Thus, AD can provoke impairments of cardiovascular functions (Yang et al., 2020), and cardiovascular diseases such as neural tissue loss‐induced stroke can also be a risk factor for AD (Breijyeh & Karaman, 2020). Hcy can directly or indirectly induce increase in oxidative stress, inflammation, and decrease in nitric oxide bioavailability. To be specific, HHcy, oxidative stress, and inhibition of nitric acid inhibition in the cell cause a cascade of reactions that can increase levels of inflammatory markers and levels of cholesterol, activity of HMG‐CoA reductase, and triglyceride and LDL that contribute to cardiovascular diseases (Sharma et al., 2015). Moreover, elevated Hcy level has been discovered and confirmed to be associated with AD (Sharma et al., 2015). Hcy was revealed to aggravate Aβ and tau lesions by elevating γ‐secretase and CDK5 activities (Li et al., 2014) and through Aβ/fibrinogen interaction (Chung et al., 2016). It was also reported that Hcy induces tau hyperphosphorylation by regulating tau kinases and phosphatases (Xia et al., 2014). HHcy induces oxidative stress, cognitive impairments, tau hyperphosphorylation, increase in calpain activity, and neurodegeneration in Sprague–Dawley rats (Mahaman et al., 2018). Hui Wei et al. have established an AD‐like rat model by injection of Hcy (400 μg/kg/day) for 14 days via the vena caudalis (Zhang et al., 2008), and Hcy‐treated rats showed typical AD‐like spatial learning and memory deficits, synaptic impairment and loss, tau hyperphosphorylation, and Aβ overload, which indicated that Hcy‐treated Sprague–Dawley rat is a suitable model for AD studies (Mahaman et al., 2018; Zeng et al., 2019; Zhang et al., 2019). The superiority of this model is that Hcy can simultaneously affect phosphorylation of the neural tau‐protein (Persson et al., 2014) and the occurrence of amyloid plaques in blood vessels (Choe et al., 2014) and in brain parenchyma (Kovalska et al., 2018; Li et al., 2014). In the current study, Hcy was used to establish a rat model for AD.

For AD diagnosis and treatment, drug administration of donepezil, galantamine, rivastigmine, and tacrine can only alleviate symptoms of AD to retard the pathological progression (Yang et al., 2020). Recently, increasing studies have been done to investigate other potential therapeutic methods. Molecular subtyping of AD corresponding to dysregulated pathways, including Aβ neuroinflammation, synaptic signaling, tau‐mediated neurodegeneration, mitochondria organization, immune activity, and myelination, have been widely investigated (Neff et al., 2021). Recently, microRNAs have been proposed to regulate cardiovascular diseases and neurodegenerative diseases (Bernardo et al., 2015; Ferrante & Conti, 2017; Mishra et al., 2016). miRNAs are small, endogenous noncoding RNAs (ncRNAs) with 21−25 nucleotides in length, which are capable of posttranscriptionally regulating gene expression by binding with 3′‐untranslated region (UTR) of mRNAs (Cao & Zhen, 2018; Ferrante & Conti, 2017; Lu & Rothenberg, 2018; Tiwari et al., 2018; Tutar, 2014). Interestingly, several serum miRNAs were identified to be aberrantly expressed in AD patient, and these dysregulated miRNAs play critical roles in the pathogenesis or the development of AD (Chang et al., 2017; Maoz et al., 2017; Swarbrick et al., 2019; Zetterberg & Burnham, 2019). In detail, miR‐124 was reported to modulate synaptic and memory impairment by affecting protein tyrosine phosphatase nonreceptor type 1 (PTPN1) signal pathway in AD (Wang et al., 2018). In addition, miR‐15/107 was proved to regulate cyclin‐dependent kinase 5 regulatory subunit 1 (CDK5R1)/p35 in the pathogenesis of AD (Parsi et al., 2015). Importantly, serum miR‐483‐3p level was identified to be downregulated in AD patients, serving as a noninvasive biomarker for AD diagnosis (Tan et al., 2014). Previously, miR‐483‐3p was widely reported to act as a tumor suppressor in a variety of tumors like breast cancer, hepatocellular carcinoma, and neuroblastoma (Huang & Lyu, 2018; Lupini et al., 2016; Wu et al., 2018). However, the biological role and potential mechanism of miR‐483‐3p in AD remain uncharacterized. Hence, the study was performed to explore the functions of miR‐483‐3p in AD and its regulatory role at the posttranscriptional level.

Exportin 1 (XPO1), also known as chromosome region maintenance protein 1 (CRM1), encodes a protein that mediates leucine‐rich nuclear export signal (NES)‐dependent protein transport (Ederle et al., 2018; Evans et al., 2018; Tian et al., 2019). Additionally, XPO1 has been reported to regulate neurodegeneration in models of frontotemporal dementia and amyotrophic lateral sclerosis (Archbold et al., 2018; Ederle et al., 2018; Steyaert et al., 2018). Furthermore, XPO1 exerted effects on cell death in traumatic brain injury (Tajiri et al., 2016; Yalçınkaya et al., 2013). Nevertheless, the role of XPO1 in AD is still unclear.

In our program, a Hcy‐induced AD rat model and Aβ1‐40‐induced cell model were established to explore the role of miR‐483‐3p and its target genes. This study may provide a potential target for the diagnosis or treatment of AD.

## 2 MATERIALS AND METHODS

### 2.1 Animals grouping and ethics statement

Total 50 adult Sprague–Dawley rats (8 weeks, male, 220–250 g) purchased from Vital River Co. Ltd. (Beijing, China) were divided into 5 groups (*n* = 10 in each group) including sham, Hcy, AAV‐miR‐483‐3p, Hcy + AAV‐NC, and Hcy + AAV‐miR‐483‐3p groups. All animal experimental procedures were approved by Institutional Animal Care and Use Committee of Hubei Provincial Hospital of Traditional Chinese Medicine. Animals were operated under the Guide for the Care and Use of Laboratory Animals of Hubei Provincial Hospital of Traditional Chinese Medicine. Timeline of the animal experiments was provided in Supporting Figure 1a.

### 2.2 The establishment of AD model

The rats (5 in each cage) were housed under 12 h reversed light‐dark cycle with free diet in a constant room temperature (22±2°C) for a week for adaption. Hcy was dissolved in saline containing 0.9% NaCl to 400 μg/ml. Rats were treated with Hcy (400 μg/kg) via the tail vein injection each day for continual 14 days. The sham‐operated rats were injected with same doze of saline.

### 2.3 Adeno‐associated virus (AAV) injection

AAV (serotype 2, 10^11^ vg/ml, Vigene Biosciences, shanghai, China) comprising miR‐483‐3p sequence was injected into Hcy‐treated rats via tail vein on day 15. The specific AAV can infect brain through tail vain injection, thereby overexpressing miR‐483‐3p in hippocampal tissues. The AAV vector was loaded in a 1 ml syringe attached to a 30‐gauge needle. Before AAV injection, rats were anesthetized under isoflurane. After a rat was placed on a heating pad, isopropanol was used to swab the tail to visualize the tail veins. The AAV was injected to one of lateral tail veins over a period of approximately 5 s per injection (Jackson et al., 2015).

### 2.4 Morris water maze (MWM) test

The Morris water maze (MWM) test was performed to measure spatial learning and memory abilities as described earlier (Vorhees & Williams, 2006). In brief, rats were allowed to swim for 1 min in the pool. Rats were trained to find the hidden platform within 1 min in each trail. The rats will be guided to stand on platform for 30 s if they failed. The training was conducted for consecutive 5 days (day 41–45, 3 trails/day). The escape latency during training days was recorded. The evaluation of spatial memory was carried out on day 46. We removed the platform and recorded the escape latency and the time spend in the target quadrant using Noldus video tracking system (Noldus Information Technology, Holland).

### 2.5 Contextual fear test

The contextual fear test was implemented to examine rat cognitive function as described previously (Wei et al., 2020a). At first, rats were placed in a contextual chamber box (32 × 26 × 26 cm) for an adaption period (3 min). Then, rats were treated with 2 footshocks (0.5 mA, 2 s) with an interval of 2 min. The percentage of freezing within 2 min was recorded. The rats were then returned to the cage. After 24 h, rats were placed in the same chamber box without footshock for fear memory ability evaluation. The total freezing time of rats was recorded.

### 2.6 Quantitative real‐time RT‐PCR

The extraction of total RNA from cultured PC12 cells and hippocampus was performed using TRIzol reagent (Invitrogen). Subsequently, the extracted RNA was reverse transcribed into cDNA using a Reverse Transcription Kit (Toyobo, Japan). The RT‐qPCR was performed using SYBR Green PCR Master Mix (Takara, Japan) with Roche Real‐Time PCR system (Roche, Basel, Switzerland). The relative RNA level was calculated with 2^−ΔΔCt^ method and normalized to GAPDH/U6. GAPDH was endogenous control for mRNAs while U6 was that for miR‐483‐3p.

### 2.7 Western blot

The total proteins of rat hippocampus and PC12 cells were extracted (Beyotime Biotechnology, China). Later, the protein samples were subjected to sodium dodecyl sulfate–polyacrylamide gel electrophoresis (SDS–PAGE), and then transferred onto a polyvinylidene fluoride (PVDF) membrane. Then, the membrane was coated with 5% defatted milk for 2 h and incubated with antibodies against XPO1 (1:1000, ab191081), APP (1:1000, ab101492), BACE1 (1:500, ab183612), Aβ1‐42 (1:1000, ab10148), and GAPDH (1:1000, ab9485) at 4°C overnight, and then incubated with HRP‐conjugated secondary antibody at room temperature for 2 h. The antibodies were purchased from Abcam (Cambridge, USA). The protein bands were examined by the Gel Image Analysis System and quantified by ImageJ software (National Institute of Health, USA).

### 2.8 Cell culture and treatment

PC12 cells (Shanghai Institute for Cell Research, shanghai, China) were grown in Dulbecco's modified Eagle's medium (DMEM, Gibco, USA) containing 10% fetal bovine serum (FBS) and 1% penicillin/streptomycin (Sigma‐Aldrich, USA) at 37°C in a humidified atmosphere with 95% air and 5% CO_2_. For cell treatment, Aβ1‐40 oligomers (2.5, 5, and 10 μM, Sigma‐Aldrich) were added.

### 2.9 Cell transfection

The full length of XPO1 was cloned to pcDNA3.1 vector to overexpress XPO1 (XPO1) with empty pcDNA3.1 vector as the negative control. MiR‐483‐3p mimics (miR‐483‐3p) were adopted to overexpress miR‐483‐3p with NC mimics as the negative control. All plasmids were synthesized by GeneChem (Shanghai, China) and transfected into PC12 cells utilizing Lipofectamine 2000 (Invitrogen, USA). After 48 h, RT‐qPCR was performed to evaluate transfection efficiency by detecting gene expression after transfection.

### 2.10 Terminal deoxynucleotidyltransferase dUTP nick end labeling (TUNEL) assay

The apoptosis of PC12 cells were detected by the In Situ Cell Death Detection kit (Roche Applied Science, Germany). The number of TUNEL positive cells and total cells at five random fields was counted. The nuclei of TUNEL positive cells would be labeled with TUNEL (green) and DAPI (blue) was used to stain those of all cells. The positive cells were counted under the fluorescence microscope (Olympus, Tokyo, Japan) using image‐Pro Plus 6.0 software.

### 2.11 TUNEL staining of hippocampus

The brain of rats was collected using 0.1 M phosphate‐buffered saline (PBS, pH 7.4) and 4% paraformaldehyde in 0.1 MPB (pH 7.4) after anesthesia with pentobarbital sodium in an In Situ Cell Death Detection Kit (Roche) as described previously (Shi et al., 2018). The hippocampus was also isolated, fixed for 48 h, and then dehydrated in alcohol. Afterward, slides were treated with xylene and washed with ethanol and distilled water twice, following the incubation with protease K for 15−30 min and washing with 1% Triton X‐100 for 8 min. Subsequently, slides were analyzed with a light microscope (Olympus).

### 2.12 Cell viability assay

PC12 cells were grown in 96‐well plates (3 × 10^4^ cells/well) for incubation overnight. Then, PC12 cells were treated with different concentrations of Aβ1‐40 or/and transfected with indicated plasmids. Following 24 h of incubation, methyl thiazolyl tetrazolium (MTT) solution (10 μl, 5 mg/ml) was added to each well for another 4 h of incubation at 37°C. After the removing culture medium, DMSO (100 μl) was added, and the absorbance was measured with a microplate reader (Bio‐Tek Instruments, USA) at 570 nm. At last, cell viability was presented as a percentage of the value in experimental groups against the corresponded control groups.

### 2.13 Luciferase reporter assay

The wild‐type (Wt) and mutant (Mut) binding site of XPO1 3′ UTR was respectively subcloned into pmirGLO vector (GeneChem) to construct XPO1‐Wt/Mut. Then, XPO1‐Wt or XPO1‐Mut was cotransfected with NC mimics or miR‐483‐3p mimics into PC12 cells using Lipofectamine 2000 (Invitrogen). The luciferase activity was evaluated by a Dual‐Luciferase Assay System (Promega, Madison, WI, USA) 48 h after plasmid transfection.

### 2.14 Statistical analysis

The data were analyzed by SPSS 19.0 software (IBM, Armonk, NY, USA) and are shown as the mean ± standard deviation (SD). The difference between two groups or among more than two was analyzed by unpaired Student's *t*‐test or one‐way ANOVA followed by Tukey's post hoc analysis. Pearson's correlation analysis was adopted to analyze the expression correlation between miR‐483‐3p and XPO1 in 20 hippocampal tissues. A *p* value less than .05 had statistical significance.

## 3 RESULTS

### 3.1 MiR‐483‐3p is downregulated in Hcy‐treated rats

**FIGURE 1:**
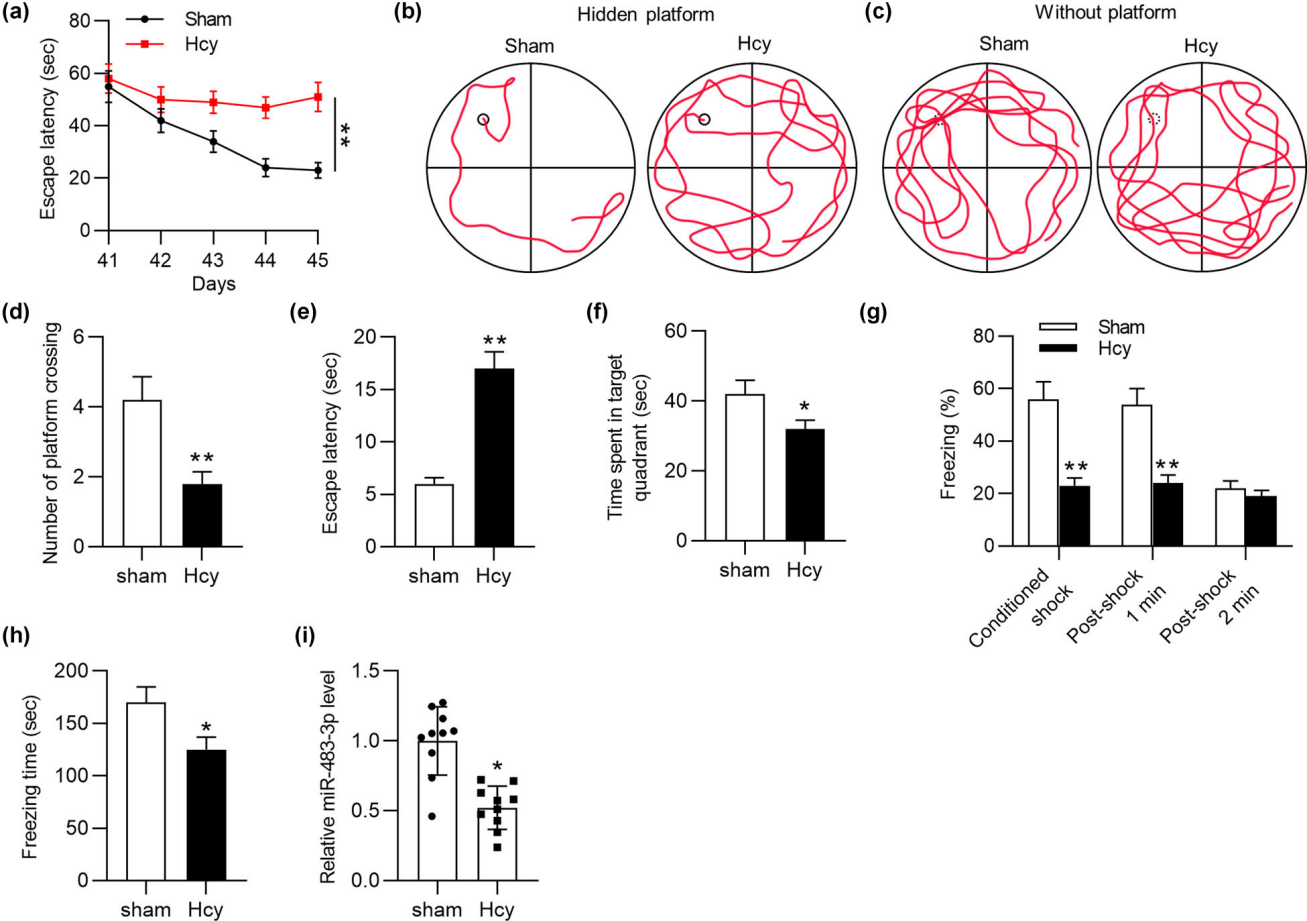
MiR‐483‐3p is downregulated in Hcy‐treated rats. (a) The escape latency of sham‐operated rats and Hcy‐treated rats during the training from day 41 to day 45. (b), (c) Swimming tracks of rats during the training and in Morris water maze (MWM) test. (d) The number of platform crossings in each group after removing the platform. (e) The escape latency of rats in MWM test. (f) After removing the platform, the time that rats spent in the target quadrant. (g) The percentage of freezing after the footshock in each group. (h) The assessment of fear memory of rats in contextual fear test. (i) The miR‐483‐3p level in hippocampus of sham‐operated rats and Hcy‐treated rats. *n* = 10/group. ^*^ *p* < .05, ^**^ *p* < .01 compared with sham group

Previously, miR‐483‐3p was reported to be downregulated in AD patients’ serum and serve as a biomarker for AD (Tan et al., 2014). However, the specific function or potential mechanism of miR‐483‐3p in AD was unclear. To figure out the role of miR‐483‐3p in AD, Hcy was injected into rats per day for consecutive 14 days to mimic AD in vivo. According to MWM test, the escape latency of Hcy model rats was significantly prolonged compared with that of rats in sham‐operated group on days 44 and 45 (Figure 1a). During the training from day 41 to day 45, swimming tracks of rats were recorded. Hcy‐treated rats had a worse performance in founding the hidden platform than control rats (Figure 1b). After the platform was removed, Hcy‐treated rats swam randomly through the tank and seldom swam across the place where the hidden platform previously been, which suggested their poor memory retention of the platform's location (Figure 1c, d). In addition, compared with rats in the sham group, the model rats had longer escape latency and spent less time in the target quadrant (Figure 1e, f). Moreover, the result of contextual fear test delineated that Hcy treatment significantly decreased the percentage of freezing and total freezing time of rats (Figure 1g, h). All these experimental data suggested that an AD‐like animal model was successfully established. Moreover, the RT‐qPCR analysis demonstrated that miR‐483‐3p was downregulated in hippocampus of Hcy‐treated rats compared with sham‐operated rats (Figure 1i). In conclusion, miR‐483‐3p level was downregulated in Hcy‐treated rat hippocampus.

### 3.2 MiR‐483‐3p overexpression alleviates impairments of learning ability, memory ability, and neural apoptosis in Hcy‐treated rats

**FIGURE 2:**
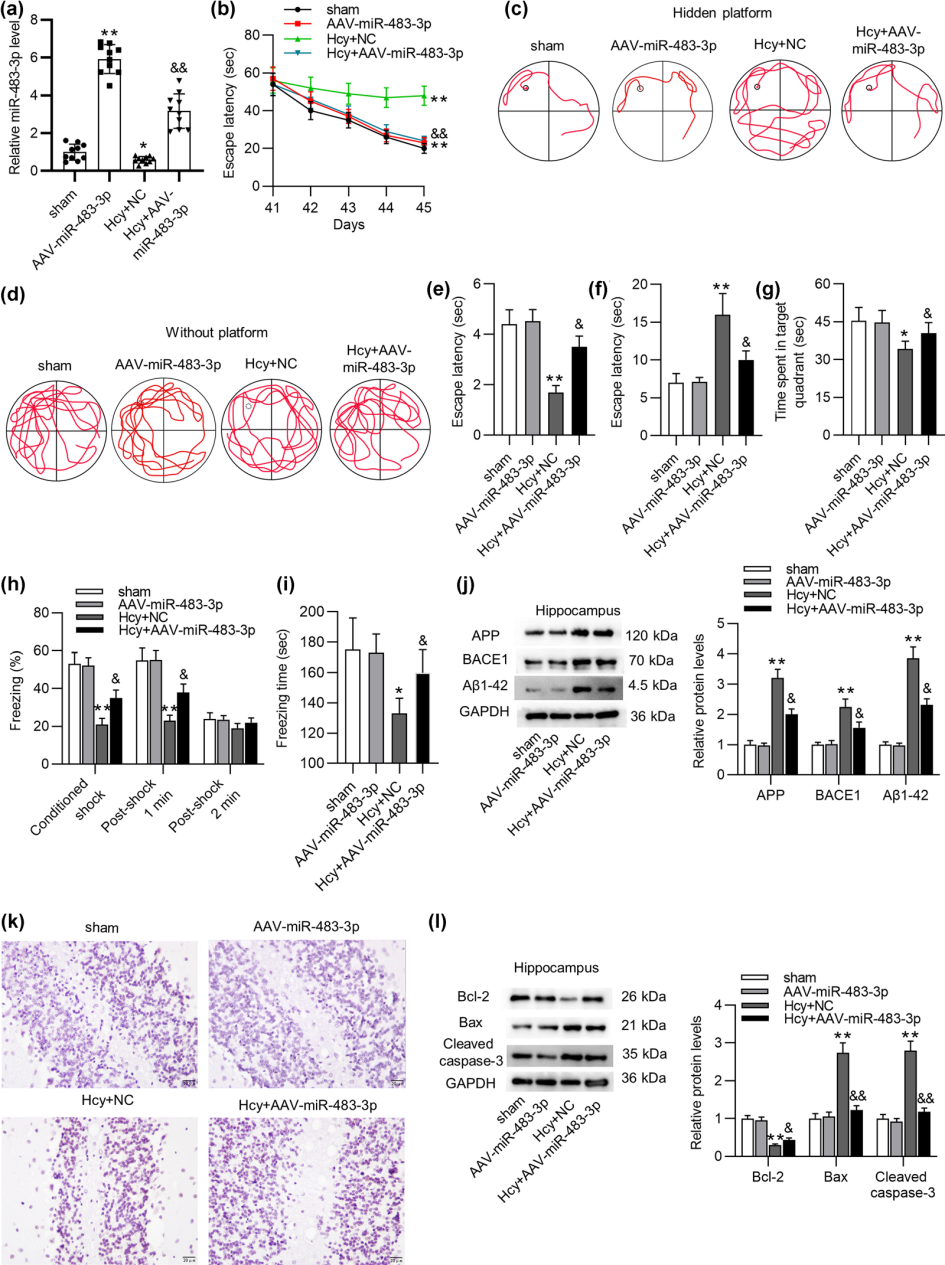
MiR‐483‐3p alleviates impairments of learning ability, memory ability, and neural apoptosis in homocysteine‐treated rats. (a) The efficacy of AAV‐miR‐483‐3p in hippocampus of rats was detected by PCR. (b) The escape latency of rats in sham group, AAV‐miR‐483‐3p, Hcy+NC group, and Hcy+AAV‐miR‐483‐3p group during the training was recorded. (c), (d) The swimming track of rats during the training and in MWM test. (e) The number of platform crossings after removing the platform. (f), (g) The escape latency and time of rats spent in the target quadrant in MWM test. (h) The percentage of freezing after footshock. (i) Fear conditioning test for contextual fear memory assessment in rats. (j) The protein levels of APP, BACE1 and Aβ1‐42 in hippocampus of rats were analyzed by western blot. (k) TUNEL assay was conducted to assess apoptosis in hippocampus of rats in the sham, AAV‐miR‐483‐3p, Hcy+NC, and Hcy+AAV‐miR‐483‐3p groups. (l) Protein levels of apoptosis markers (Bcl‐2, Bax, and cleaved caspase‐3) in hippocampus of rats were quantified using western blot. *n* = 10/group. ^*^ *p* < .05, ^**^ *p* < .01 compared with sham group; ^&^ *p* < .05, ^&&^ *p* < .01 compared with Hcy group

We then evaluated the effects of miR‐483‐3p on learning and memory abilities of Hcy‐treated rats. At first, miR‐483‐3p level in hippocampus of sham‐operated or Hcy‐treated rats was significantly overexpressed by injection of AAV‐miR‐483‐3p (Figure 2a). Additionally, MWM test suggested that miR‐483‐3p significantly shortened the escape latency of Hcy rats during training (Figure 2b). Swimming tracks also revealed that rats in the Hcy+AAV‐miR‐483‐3p group had better performance than those in the Hcy+NC group during training (Figure 2c). In the test, Hcy model rats with AAV‐miR‐483‐3p adopted a spatially biased search strategy to locate the platform, implying good memory retention (Figure 2d). The number of platform crossings also demonstrated the increase in memory retention mediated by AAV‐miR‐483‐3p (Figure 2e). Moreover, miR‐483‐3p overexpression shortened the escape latency of Hcy‐treated rats (Figure 2f). Meanwhile, rats injected with AAV‐miR‐483‐3p spent more time in target quadrant than Hcy‐treated rats (Figure 2g). These results confirmed that miR‐483‐3p overexpression improved the spatial learning and memory ability of model rats. Afterward, we implemented contextual fear test to evaluate the contextual fear memory of rats. The percentage of freezing during training and the total freezing time in the test were prolonged by miR‐483‐3p overexpression (Figure 2h, i), suggesting miR‐483‐3p overexpression alleviated the impairment of fear memory resulting from Hcy treatment. Western blot analysis demonstrated that Hcy‐mediated the increase in protein levels of AD‐related factors including APP, BACE1, and Aβ1‐42 was prominently reversed by miR‐483‐3p overexpression (Figure 2j). Figure 2k revealed that miR‐483‐3p rescued the Hcy‐induced apoptosis in hippocampus of rats. Protein levels of apoptotic markers (Bcl‐2, Bax, and Cleaved caspase‐3) in hippocampus tissues were quantified using western blot. As suggested by Figure 2l, overexpressed miR‐483‐3p rescued the decrease in Bcl‐2 protein level while reversing the upregulation of Bax and cleaved caspase‐3 levels. Collectively, miR‐483‐3p alleviated impairments of learning and memory abilities as well as suppressed brain injury in Hcy‐treated rats.

### 3.3 MiR‐483‐3p overexpression inhibits the apoptosis of Aβ1‐40‐stimulated PC12 cells and downregulates levels of AD‐associated proteins

**FIGURE 3:**
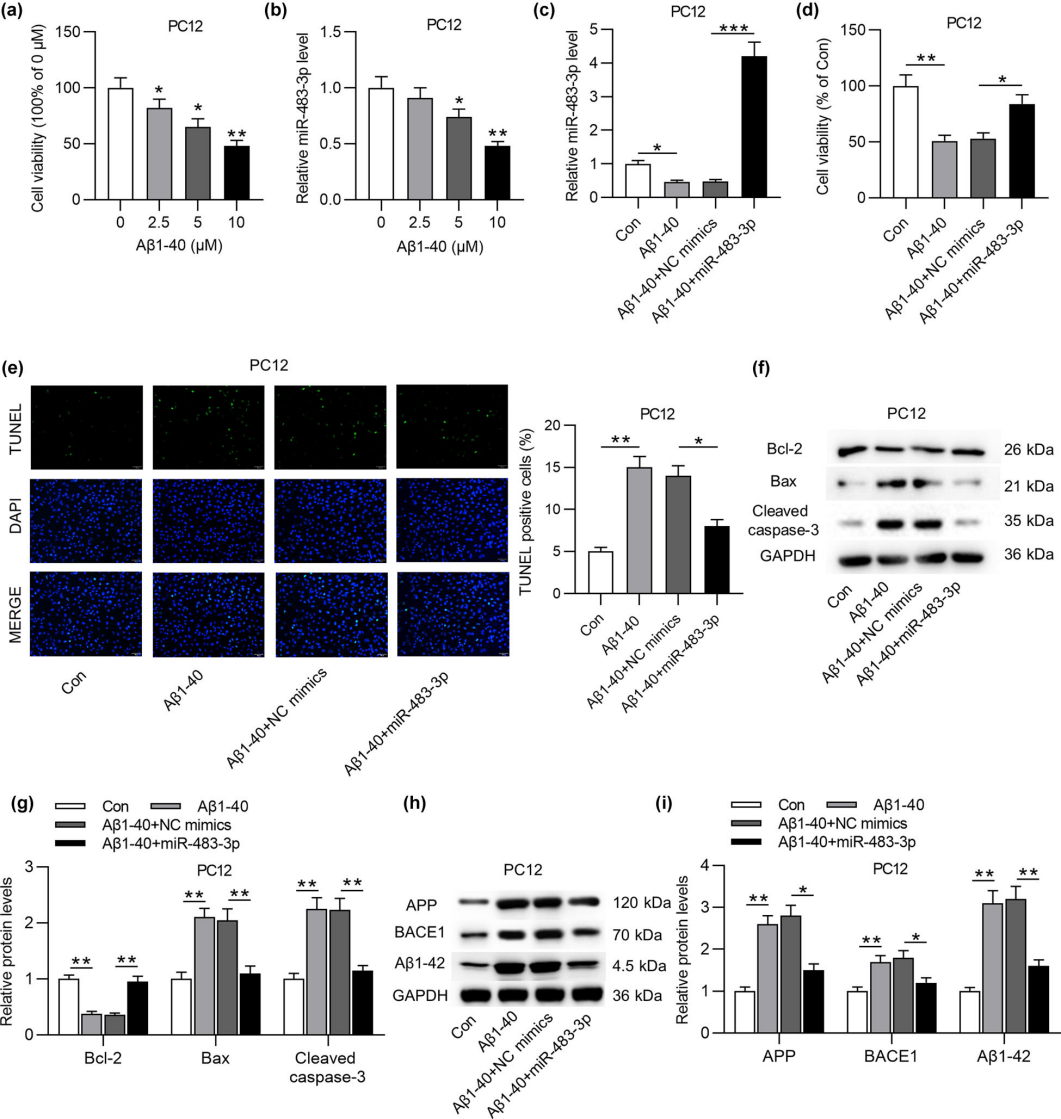
MiR‐483‐3p overexpression inhibits the apoptosis of Aβ1‐40‐stimulated PC12 cells and downregulates levels of AD‐associated proteins. (a) The viability of PC12 cells in response to Aβ1‐40 treatment (2.5, 5, or 10 μM) was evaluated by MTT assay. (b) The miR‐483‐3p level in PC12 cells treated with different concentrations of Aβ1‐40 was analyzed by RT‐qPCR. (c) The miR‐483‐3p level in control group, Aβ1‐40 (5 μM) group, Aβ1‐40 (5 μM) + NC mimics group, and Aβ1‐40 (5 μM) + miR‐483‐3p group was analyzed by RT‐qPCR. (d) The viability of PC12 cells in above four groups was evaluated by MTT assay. (e) The apoptosis of PC12 cells in above four groups was measured by TUNEL assay. (f), (g) Protein levels of apoptotic markers (Bcl‐2, Bax, and cleaved caspase‐3) in PC12 cells with above transfection and treatment were quantified using western blot. (h), (i) APP, BACE1, and Aβ1‐42 protein levels in PC12 cells of above four groups were examined by western blot analysis. ^*^ *p* < .05, ^**^ *p* < .01, ^***^ *p* < .001

To figure out the role of miR‐483‐3p in vitro, PC12 cells were treated with different concentrations of Aβ1‐40 to induce neuron injury. As displayed in Figure 3a, MTT assay showed that the increasing concentration of Aβ1‐40 induced the gradual decrease of PC12 cell viability. In addition, the high concentration (5 or 10 μM) of Aβ1‐40 treatment also triggered a decline of miR‐483‐3p level in PC12 cells (Figure 3b). For subsequent experiments, 5 μM Aβ1‐40 was used to treat PC12 cells. Next, miR‐483‐3p was overexpressed by transfecting miR‐483‐3p mimics into PC12 cells (Figure 3c). Based on MTT assay, miR‐483‐3p overexpression antagonized the suppressive effect of Aβ1‐40 on the viability of PC12 cells (Figure 3d). Inversely, Aβ1‐40‐induced the enhancement of cell apoptosis was reversed by miR‐483‐3p overexpression (Figure 3e). Additionally, Aβ1‐40 induced downregulation of Bcl‐2 protein level while upregulating Bax and cleaved caspase‐3 levels (Figure 3f, g). After transfection of miR‐483‐3p mimics into Aβ1‐40‐treated PC12 cells, the decrease in Bcl‐2 protein level and the increase in Bax and cleaved caspase‐3 levels induced by Aβ1‐40 were partially reversed by overexpressed miR‐483‐3p (Figure 3f, g). At last, the upregulation of APP, BACE1 and Aβ1‐42 protein levels resulting from Aβ1‐40 treatment was neutralized by miR‐483‐3p overexpression (Figure 3h, i). In conclusion, miR‐483‐3p inhibits the apoptosis of Aβ1‐40‐stimulated PC12 cells and downregulates levels of AD‐associated proteins in cells.

### 3.4 MiR‐483‐3p targets XPO1 by binding with its 3´ UTR

**FIGURE 4:**
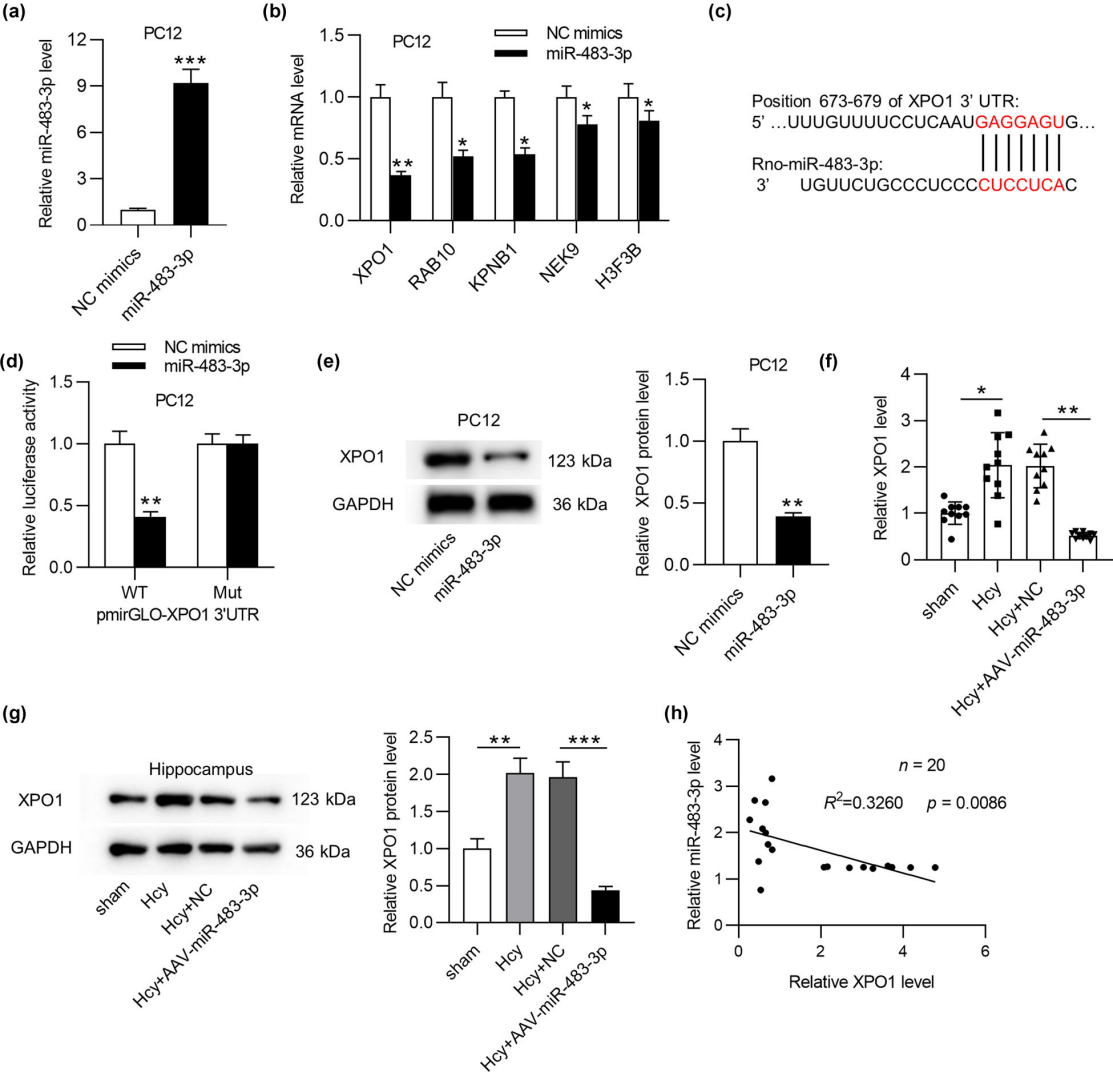
MiR‐483‐3p targets XPO1 by binding with its 3´ UTR. (a) The overexpression efficiency of miR‐483‐3p in PC12 cells was detected by RT‐qPCR. (b) The mRNA levels of predicted mRNAs in the context of miR‐483‐3p overexpression were measured by RT‐qPCR. (c) The binding sequences between miR‐483‐3p and XPO1 3´ UTR. (d) The luciferase reporter assay was adopted to evaluate the binding relation between miR‐483‐3p and XPO1 3´ UTR. (e) The protein level of XPO1 in PC12 cells was detected by western blot analysis. (f), (g) The mRNA and protein levels of XPO1 in hippocampus of rats in Hcy, Hcy+NC, and Hcy+AAV‐miR‐483‐3p group (*n* = 10/group) were detected by PCR and western blot. (h) The expression correlation between miR‐483‐3p and XPO1 in hippocampus of Hcy‐treated rats (*n* = 20) was analyzed by Pearson's correlation analysis. ^*^ *p* < .05, ^**^ *p* < .01, ^***^ *p* < .001

Growing studies proposed that miR‐483‐3p could regulated gene expression at the posttranscriptional level by binding with 3´ UTR of target mRNA, thus we hypothesized that miR‐483‐3p also acted similarly in AD. To begin with, miR‐483‐3p level was prominently overexpressed by transfection of miR‐483‐3p mimics in PC12 cells (Figure 4a). To predict the potential target mRNA(s) of miR‐483‐3p, we searched starBase website (http://starbase.sysu.edu.cn/), and 15 mRNAs were screened out (supplementary Table 1). Additionally, data from RT‐qPCR analysis disclosed that 5 mRNAs (XPO1, RAB10, KPNB1, NEK9, and H3F3B) showed the significant downregulation in response of miR‐483‐3p overexpression (Figure 4b) while the rest mRNAs displayed no significant alteration (supporting Figure 1b). Moreover, XPO1 indicated the most downregulation among these 5 mRNAs in the context of miR‐483‐3p overexpression. Furthermore, XPO1 has been identified to be involved in the regulation of several neurodegenerative diseases including amyotrophic lateral sclerosis and frontotemporal dementia (Archbold et al., 2018; Ederle et al., 2018; Steyaert et al., 2018). Hence, XPO1 was chosen for further exploration. According to Targetscan (http://www.targetscan.org/), the binding sequences between miR‐483‐3p and XPO1 were shown in Figure 4c. Then, the luciferase reporter assay disclosed that miR‐483‐3p mimics obviously reduced the luciferase activity of XPO1‐WT vector, and no significant change was observed in XPO1‐Mut vector (Figure 4d). Likewise, miR‐483‐3p mimics also triggered an apparent reduction of XPO1 protein level (Figure 4e). Furthermore, XPO1 mRNA and protein levels were upregulated in hippocampus of Hcy‐treated rats compared with those in sham‐operated rats’ hippocampus, and miR‐483‐3p overexpression induced the downregulation of XPO1 mRNA expression in Hcy model rats (Figure 4f, g). Finally, Pearson's correlation analysis demonstrated that XPO1 expression was negatively correlated with miR‐483‐3p expression in hippocampus of Hcy‐induced AD‐like rats (Figure 4h). In summary, miR‐483‐3p inhibited XPO1 by binding with its 3´ UTR.

### 3.5 Silencing XPO1 inhibits the apoptosis of Aβ1‐40‐stimulated PC12 cells and downregulates levels of AD‐associated proteins

**FIGURE 5:**
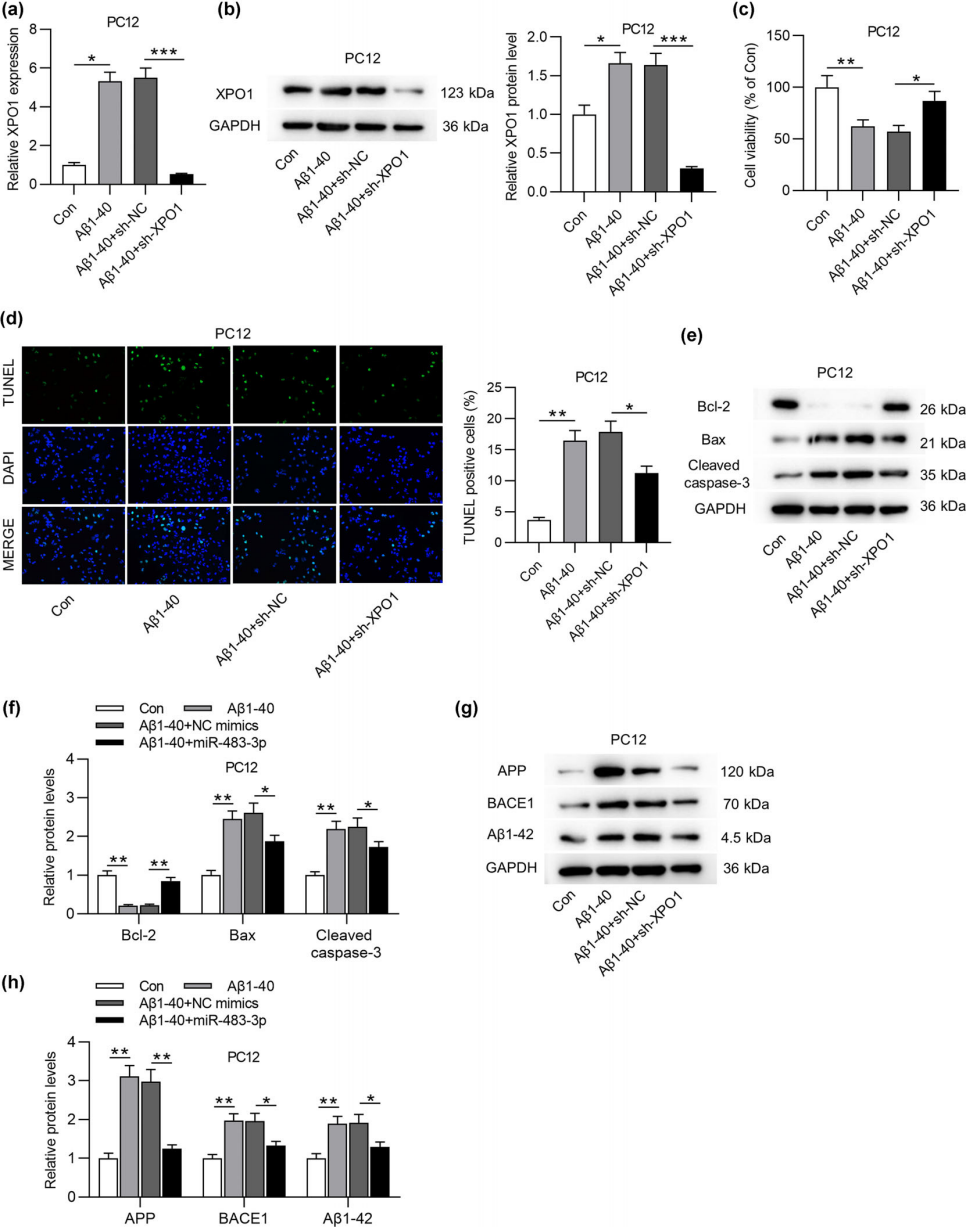
Silencing XPO1 inhibits the apoptosis of Aβ1‐40‐stimulated PC12 cells and downregulates levels of AD‐associated proteins. (a), (b) The expression levels of XPO1 in Aβ1‐40‐treated PC12 cells and its knockdown efficacy after transfection of sh‐XPO1 were detected by PCR and western blot. (c), (d) The viability and apoptosis of PC12 cells in four groups (Con, Aβ1‐40, Aβ1‐40+sh‐NC, and Aβ1‐40+sh‐XPO1) were examined by MTT and TUNEL assays. (e)–(h) Protein levels of apoptosis markers (Bcl‐2, Bax, and Cleaved caspase‐3) and AD‐associated factors (APP, BACE1, and Aβ1‐42) in PC12 cells of above four groups were quantified utilizing western blot. ^*^ *p* < .05, ^**^ *p* < .01, ^***^ *p* < .001

Since the role of XPO1 in AD has not been reported, we explored the role of XPO1 in regulating neural cell apoptosis and protein levels of AD‐related factors in cells. RT‐qPCR and western blot analyses revealed upregulation of XPO1 in PC12 cells after Aβ1‐40 treatment and the successful knockdown efficacy of XPO1 was implied in Aβ1‐40+sh‐XPO1 group (Figure 5a, b). Silencing XPO1 rescued the decrease in the viability of Aβ1‐40‐treated PC12 cells and reversed the enhancement of cell apoptosis, as suggested by the MTT assay and TUNEL assay (Figure 5c, d). In addition, XPO1 deficiency countervailed the reduction of Bcl‐2 protein level and the enhancement of Bax and Cleaved caspase‐3 protein levels (Figure 5e, f). Moreover, the levels of AD related proteins (APP, BACE1, and Aβ1‐42) were upregulated by Aβ1‐40 treatment and partially reversed by XPO1 depletion (Figure 5g, h).

### 3.6 XPO1 overexpression reverses the suppressive effect of miR‐483‐3p on cell apoptosis and protein levels of AD‐associated factors

**FIGURE 6:**
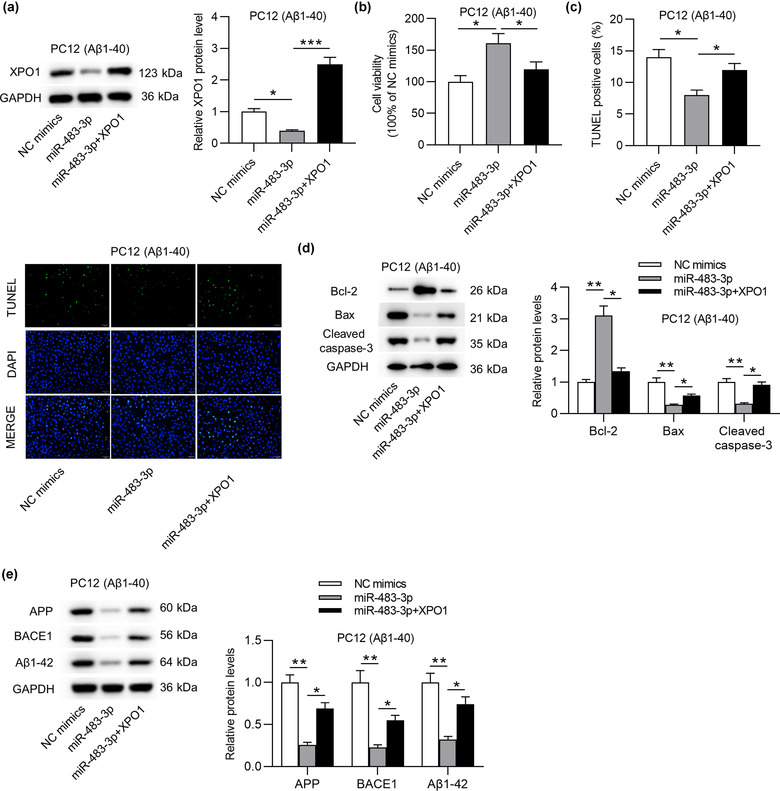
XPO1 overexpression reverses the suppressive effect of miR‐483‐3p on cell apoptosis and protein levels of AD‐associated factors. (a) The overexpression efficacy of XPO1 in the context of miR‐483‐3p overexpression was validated by western blot analysis. (b), (c) The effects of overexpressing miR‐483‐3p and XPO1 on the viability and apoptosis of PC12 cells were examined by MTT assay and TUNEL assay. (d), (e) The effects of overexpressed XPO1 on apoptosis markers and APP, BACE1, and Aβ1‐42 protein levels in response to miR‐483‐3p overexpression were determined by western blot. ^*^ *p* < .05, ^**^ *p* < .01, ^***^ *p* < .001

To further validate that miR‐483‐3p regulated cell apoptosis and AD‐associated proteins by targeting XPO1, rescue assays were implemented. Initially, XPO1 protein level was downregulated by miR‐483‐3p overexpression and then successfully overexpressed by transfection of pcDNA3.1/XPO1 into Aβ1‐40‐stimulated PC12 cells (Figure 6a). In addition, MTT assay indicated that miR‐483‐3p mimics‐mediated the rise of cell viability was offset by XPO1 overexpression (Figure 6b). On the contrary, the reduction of cell apoptosis resulting from miR‐483‐3p overexpression was antagonized by XPO1 overexpression (Figure 6c). Upregulated Bcl‐2 protein level and downregulated Bax and cleaved caspase‐3 protein levels induced by overexpressing miR‐483‐3p were partially reversed by XPO1 overexpression (Figure 6d). In the end, the suppressive effect of miR‐483‐3p mimics on levels of AD‐associated proteins was offset by XPO1 overexpression (Figure 6e). In a word, miR‐483‐3p inhibits cell apoptosis and levels of AD‐associated proteins by targeting XPO1.

## 4 DISCUSSION

It is well established that the cognitive impairment and memory decline are typical characteristics of AD, which are strongly correlated with Aβ plaque accumulation, neuron apoptosis, and neurofibrillary tangle formation (Lim et al., 2018; Roberts et al., 2016). According to previous studies, Aβ results in synaptic spine shrinkage, weakens the synaptic transmission, and finally disrupts the long‐term potentiation. This predominant form of synaptic plasticity is regarded as the potential cellular mechanism of learning, cognition, and memory in the brain (Cai et al., 2014; O'Brien & Wong, 2011; Shokri‐Kojori et al., 2018; Thal et al., 2015). To explore the functions and mechanisms of molecules in neural cells, Aβ1‐40 was frequently used for establishment of in vitro cell model (Feng et al., 2013; Hu et al., 2021; Qian et al., 2015). For establishment of in vivo animal model, some previous studies were reported to use Hcy‐induced rats (Mahaman et al., 2018; Zhang et al., 2008). Hyperhomocysteinemia is regarded as an independent cause of AD (Obeid & Herrmann, 2006; Seshadri et al., 2002). Implicated in the etiology of AD, hyperhomocysteinemia influences brain parenchyma by the disorder of oxidative state and free radical‐induced damage to neuronal membrane components (Kovalska et al., 2018). Clinical studies also revealed that Hcy is associated with the degree of cognitive impairment and acts as a biomarker for the progression of AD dementia (Kitzlerová et al., 2014). In our study, we adopted Aβ1‐40‐treated PC12 cells to establish cell model and Hcy injection to induce AD rat model. We discovered that Hcy injection significantly impaired learning, cognitive, and memory abilities of model rats.

Accumulating research proposed that miRNAs are implicated in the initiation or development of AD by exerting effects on cellular processes (Chang et al., 2017; Maoz et al., 2017; Swarbrick et al., 2019; Zetterberg & Burnham, 2019). For instance, upregulated miR‐34c induces synaptic and cognitive function deficits by binding with synaptotagmin 1 via the ROS‐JNK‐p53 pathway in AD (Shi et al., 2020). In addition, miR‐181a was proven to negatively regulate synaptic plasticity and memory deficits in a mouse model of AD (Rodriguez‐Ortiz et al., 2020). Although serum miR‐483‐3p has been reported to be downregulated in AD patients (Tan et al., 2014), the role of miR‐483‐3p remains to be explored in AD. In the current study, we discovered that the impairment of learning, cognitive and memory abilities of rats induced by Hcy injection was improved by miR‐483‐3p overexpression. Considering that neuron apoptosis is related to the progression of AD (Jazvinšćak Jembrek et al., 2015; Wei et al., 2018), we also evaluated the effects of miR‐483‐3p on neuron apoptosis in rat hippocampus and found that miR‐483‐3p overexpression antagonized Hcy‐induced the enhancement of neuron apoptosis. Previously, Aβ peptides were reported to be produced by the cleavage of amyloid precursor protein (APP) influenced by γ‐secretase and β‐site APP cleaving enzyme 1 (BACE1) (Laird et al., 2005; Zhang et al., 2017). Moreover, the accumulation of Aβ peptides is believed to be the leading cause of AD. Hence, we evaluate levels of these proteins (APP, BACE1, and Aβ1‐42) in rat hippocampus, and the experimental results confirmed that overexpression of miR‐483‐3p offset the promoting effects of Hcy on these protein levels. MiR‐483‐3p overexpression also alleviated Aβ1‐40‐treated PC12 cell apoptosis and reversed the upregulation of APP, BACE1, and Aβ1‐42 protein levels in cells.

MiR‐483‐3p was reported to bind with 3′ UTR of mRNAs. Specifically, upregulated miR‐483‐3p was proposed to impair the vascular injury by binding with VEZF1 in type 2 diabetes (Kuschnerus et al., 2019). Furthermore, downregulation of miR‐483‐3p facilitates acquired gefitinib resistance and epithelial‐to‐mesenchymal transition by targeting integrin β3 in EGFR‐mutant NSCLC (Yue et al., 2018). With the assistance of starBase, 15 mRNAs possessing binding site on miR‐483‐3p were screened out, and XPO1 was validated to serve as a direct target gene of miR‐483‐3p. Moreover, miR‐483‐3p negatively regulated XPO1 protein level, and there is a negative correlation between hippocampal miR‐483‐3p and XPO1 expression in Hcy‐induced AD rats.

XPO1 plays an essential role in neurodegenerative diseases including amyotrophic lateral sclerosis and frontotemporal dementia (ALS/FTD) (Archbold et al., 2018; Ederle et al., 2018; Steyaert et al., 2018). For example, in the investigation of ALS/FTD, XPO1 inhibitors was found to reduce cell death induced by TAR DNA‐binding protein‐43 (TDP43) overexpression in cortical neurons, suggesting the neuroprotective role of XPO1 (Chou et al., 2018). In another study, XPO1 overexpression can effectively promote the export of nuclear TDP43, and high cytoplasmic TDP43 concentrations inhibit the survival of neurons (Archbold et al., 2018). In addition, XPO1 exerts its nuclear protein export function to regulate cell cycle, apoptosis, and death (Crochiere et al., 2015; Kashyap et al., 2016; Nie et al., 2018). Likewise, in the current study, XPO1 overexpression neutralized the suppressive effect of miR‐483‐3p on PC12 cell death and levels of AD associated proteins (APP, BACE1, and Aβ1‐42) in cells. XPO1 acts as an exporter of over 200 known cargos, such as p53, p21, IκB‐α, and NF‐κB (Kashyap et al., 2016). Inhibition of XPO1 may influence many inflammatory/immune pathways associated with neurological diseases, such as Nrf2, FOXOs, and NF‐κB signaling pathways (Archbold et al., 2018). In the current study, we did not explore the downstream signaling mediated by the miR‐483‐3p/XPO1 axis in AD, and more investigation will be conducted in the future.

In conclusion, miR‐483‐3p alleviates neuron apoptosis and impairments of learning and memory abilities by targeting XPO1 in AD. This discovery may provide a potential target for the diagnosis or treatment of AD. However, our study has limitations. First, the role of XPO1 in AD was not explored in vivo. Second, other potential mechanism of miR‐483‐3p in AD remained to be explored.

## COMPETING INTERESTS

The authors declare that they have no competing interests.

## Supporting information

Supplementary Table S1: Candidate target mRNAs of miR‐483‐3p.

Supporting Figure S1 (a) Timeline of animal studies. Rats (*n* = 40) were housed for adaption for 1 week. From day 1 to day 14, rats were injected with Hcy (*n* = 20) or saline (sham group, *n* = 10) once a day for consecutive 14 days. Then, Hcy‐treated rats were injected with adeno‐associated virus containing miR‐483‐3p (*n* = 10) on day 15. The MWM test training was conducted on days 41–45, and the contextual fear test and MWM test were carried out on day 46. At last, rats were sacrificed, and the serum and hippocampus of rats were collected for other experiments. (b) The mRNA levels of 10 predicted mRNAs that were not significantly influenced by miR‐483‐3p overexpression were presented, as measured by RT‐qPCR.

## Data Availability

The data sets used or analyzed during the current study are available from the corresponding author on reasonable request.
