# Supplementary material for: MiR‐483‐3p improves learning and memory abilities via XPO1 in Alzheimer's disease
Source: Brain Behav. 2022 Jul 14;12(8):e2680. doi: 10.1002/brb3.2680 (PMC9392541; doi:10.1002/brb3.2680)
Supplement: Supplementary file 1 — Supplementary Table S1: Candidate target mRNAs of miR‐483‐3p. [file BRB3-12-e2680-s002.docx]

**Supplementary table 1: Candidate target mRNAs of miR-483-3p.**

| miRNA  name | Gene ID | Gene  Name | PITA | miRmap | microT | PicTar | TargetScan |
| --- | --- | --- | --- | --- | --- | --- | --- |
| hsa-miR-483-3p | ENSG00000171314 | PGAM1 | 1 | 1 | 1 | 1 | 1 |
| hsa-miR-483-3p | ENSG00000156599 | ZDHHC5 | 1 | 1 | 1 | 1 | 1 |
| hsa-miR-483-3p | ENSG00000119638 | NEK9 | 1 | 1 | 1 | 1 | 1 |
| hsa-miR-483-3p | ENSG00000132475 | H3F3B | 1 | 1 | 1 | 1 | 1 |
| hsa-miR-483-3p | ENSG00000108424 | KPNB1 | 1 | 1 | 1 | 1 | 1 |
| hsa-miR-483-3p | ENSG00000082898 | XPO1 | 1 | 1 | 1 | 1 | 1 |
| hsa-miR-483-3p | ENSG00000084733 | RAB10 | 1 | 1 | 1 | 1 | 1 |
| hsa-miR-483-3p | ENSG00000158470 | B4GALT5 | 1 | 1 | 1 | 1 | 1 |
| hsa-miR-483-3p | ENSG00000138767 | CNOT6L | 1 | 1 | 1 | 1 | 1 |
| hsa-miR-483-3p | ENSG00000177733 | HNRNPA0 | 1 | 1 | 1 | 1 | 1 |
| hsa-miR-483-3p | ENSG00000010818 | HIVEP2 | 1 | 1 | 1 | 1 | 1 |
| hsa-miR-483-3p | ENSG00000198791 | CNOT7 | 1 | 1 | 1 | 1 | 1 |
| hsa-miR-483-3p | ENSG00000129292 | PHF20L1 | 1 | 1 | 1 | 1 | 1 |
| hsa-miR-483-3p | ENSG00000147162 | OGT | 1 | 1 | 1 | 1 | 1 |
| hsa-miR-483-3p | ENSG00000131725 | WDR44 | 1 | 1 | 1 | 1 | 1 |
