# Supplementary figures and images for: MiR‐483‐3p improves learning and memory abilities via XPO1 in Alzheimer's disease
Source: Brain Behav. 2022 Jul 14;12(8):e2680. doi: 10.1002/brb3.2680 (PMC9392541; doi:10.1002/brb3.2680)

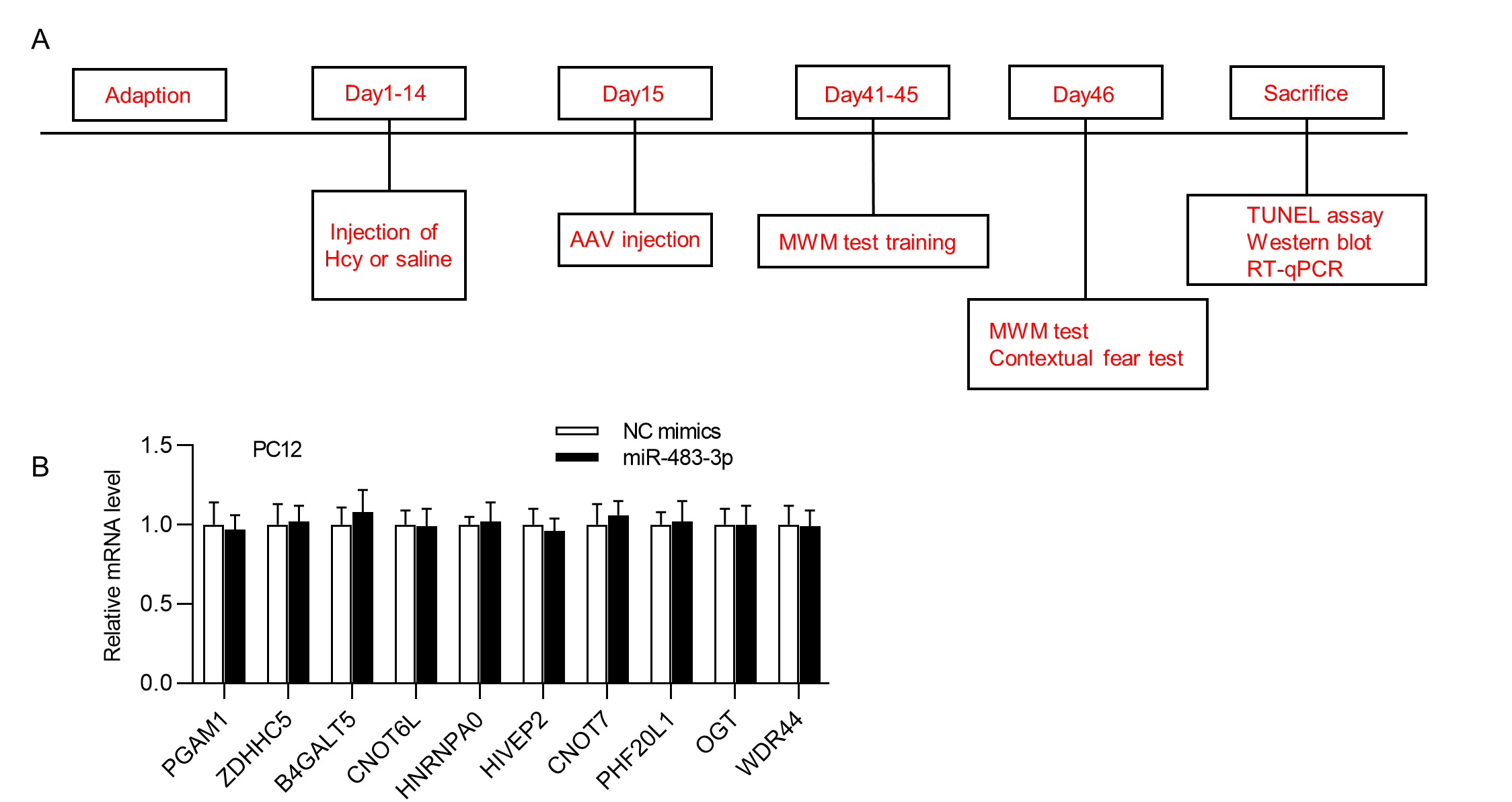

Supplement: Supplementary file 2 — Supporting Figure S1 (a) Timeline of animal studies. Rats (n = 40) were housed for adaption for 1 week. From day 1 to day 14, rats were injected with Hcy (n = 20) or saline (sham group, n = 10) once a day for consecutive 14 days. Then, Hcy‐treated rats were injected with adeno‐associated virus containing miR‐483‐3p (n = 10) on day 15. The MWM test training was conducted on days 41–45, and the contextual fear test and MWM test were carried out on day 46. At last, rats were sacrificed, and the serum and hippocampus of rats were collected for other experiments. (b) The mRNA levels of 10 predicted mRNAs that were not significantly influenced by miR‐483‐3p overexpression were presented, as measured by RT‐qPCR. [file BRB3-12-e2680-s001.TIF]
